# Ginseng extract and ginsenoside Rb1 attenuate carbon tetrachloride-induced liver fibrosis in rats

**DOI:** 10.1186/1472-6882-14-415

**Published:** 2014-10-25

**Authors:** Ya-Ling Hou, Ya-Hui Tsai, Yun-Ho Lin, Jane C-J Chao

**Affiliations:** School of Nutrition and Health Sciences, College of Public Health and Nutrition, Taipei Medical University, Taipei 110, Taiwan; School of Dentistry, Taipei Medical University, Taipei 110, Taiwan; Master Program in Global Health and Development, College of Public Health and Nutrition, Taipei Medical University, Taipei 110, Taiwan; Nutrition Research Center, Taipei Medical University Hospital, Taipei 110, Taiwan

**Keywords:** Ginsenoside Rb1, Carbon tetrachloride, Interleukin-1β, Liver fibrosis, Tissue inhibitor of metalloproteinase-1

## Abstract

**Background:**

Ginsenosides, the major bioactive compounds in ginseng root, have been found to have antioxidant, immunomodulatory and anti-inflammatory activities. This study investigated the effects of ginsenosides on carbon tetrachloride (CCl_4_)-induced hepatitis and liver fibrosis in rats.

**Methods:**

Male Sprague–Dawley rats were randomly divided into four groups: control, CCl_4_, CCl_4_ + 0.5 g/kg *Panax ginseng* extract and CCl_4_ + 0.05 g/kg ginsenoside Rb1 groups. The treated groups were orally given *Panax ginseng* extract or ginsenoside Rb1 two weeks before the induction of liver injury for successive 9 weeks. Liver injury was induced by intraperitoneally injected with 400 ml/l CCl_4_ at a dose of 0.75 ml/kg body weight weekly for 7 weeks. The control group was intraperitoneally injected with olive oil.

**Results:**

The pathological results showed that ginsenoside Rb1 decreased hepatic fat deposition (2.65 ± 0.82 *vs* 3.50 ± 0.75, *p* <0.05) and *Panax ginseng* extract lowered hepatic reticular fiber accumulation (1.05 ± 0.44 *vs* 1.60 ± 0.39, *p* <0.01) increased by CCl_4_. Plasma alanine aminotransferase and aspartate aminotransferase activities were increased by CCl_4_ (*p* <0.01)_,_ and aspartate aminotransferase activity was decreased by *Panax ginseng* extract at week 9 (*p* <0.05). Exposure to CCl_4_ for 7 weeks, the levels of plasma and hepatic triglycerides (*p* <0.01), hepatic cholesterol (*p* <0.01), interleukin-1β (*p* <0.01), prostaglandin E_2_ (*p* <0.05), soluble intercellular adhesion molecule-1 (*p* <0.05), hydroxyproline (*p* <0.05), matrix metalloproteinase-2 (*p* <0.05) and tissue inhibitor of metalloproteinase-1 (TIMP-1) (*p* <0.01) were elevated, however, hepatic interleukin-10 level was lowered (*p* <0.05). Both *Panax ginseng* extract and ginsenoside Rb1 decreased plasma and hepatic triglyceride, hepatic prostaglandin E_2_, hydroxyproline and TIMP-1 levels, and *Panax ginseng* extract further inhibited interleukin-1β concentrations (*p* <0.05).

**Conclusions:**

*Panax ginseng* extract and ginsenoside Rb1 attenuate plasma aminotransferase activities and liver inflammation to inhibit CCl_4_-induced liver fibrosis through down-regulation of hepatic prostaglandin E_2_ and TIMP-1.

## Background

Liver cirrhosis is an irreversible stage in the process of liver damage that occurs after liver fibrosis. Liver fibrosis is attributed to inflammation, excessive accumulation of extracellular matrix and tissue remodeling under wound healing [1]. Chronic hepatitis and liver cirrhosis are positively associated with the occurrence of hepatocellular carcinoma [2, 3]. Therefore, the inhibition of hepatic inflammation and fibrosis is crucial in preventing the occurrence of liver cirrhosis and hepatocellular carcinoma.

Oxidative stress from reactive oxygen species plays an important role in liver fibrogenesis [4]. Carbon tetrachloride (CCl_4_) is considered as a toxic chemical that induces hepatotoxicity including fatty degeneration, inflammation, fibrosis, hepatocellular death and carcinogenicity [5, 6]. Trichloromethyl radical produced from the metabolism of CCl_4_ initiates a chain reaction to cause lipid peroxidation, membrane dysfunction and further hepatotoxic damage [6]. The toxic metabolite of CCl_4_ can activate Kupffer cells to secrete cytokines such as interleukin-1 (IL-1) and tumor necrosis factor-α (TNF-α), stimulate transforming growth factor-β (TGF-β) production, inhibit nitric oxide (NO) formation and induce inflammation and liver fibrosis [6–8]. Matrix metalloproteinase (MMP)-2, known as type IV collagenase and gelatinase A, acts as the regulator for the breakdown of extracellular matrix, and tissue inhibitor metalloproteinase (TIMP)-1, as the inhibitor of MMPs, exhibits anti-fibrolytic, growth-stimulated and anti-apoptotic activities [9]. Chronic exposure of CCl_4_ leads to liver fibrosis, which diminishes extracellular matrix degradation and increases MMP-2 secretion through the induction of tissue inhibitor TIMPs [9].

*Panax ginseng* (*P. ginseng*) root has been commonly used in oriental medicine, diet or dietary supplement. Ginsenosides, a class of steroid glycosides and triterpene saponins, are the major bioactive compounds in *P. ginseng* root and ginsenoside Rb1 (C_54_H_92_O_23_, molecular weight: 1109.3) is considered as the most abundant ginsenoside among more than 30 ginsenosides in *P. ginseng* [10, 11]. The previous studies have reported that *P. ginseng* and its active components or metabolites had antioxidant, immunomodulatory, anti-inflammatory, and lipid-lowering effects [12–15]. Many studies have shown that ginsenoside Rb1 and its metabolite compound K attenuated liver injury through inhibiting lipid peroxidation, TNF-α, NO, prostaglandin E_2_ (PGE_2_), intercellular adhesion molecule (ICAM)-1 and nuclear factor-κB (NF-κB) activation [16–19]. However, the effect of ginsenosides on liver fibrosis is not clear. Considering ginsenoside Rb1 as the most abundant ginsenoside in *P. ginseng* [10, 11] and its hepatoprotective activity [16–19], therefore, this study investigated the protective effects of *P. ginseng* extract (ginseng extract) and ginsenoside Rb1 on CCl_4_-induced liver inflammation and fibrosis in rats.

## Methods

### Animals and treatments

Sprague–Dawley rats weighing 200–250 g were purchased from the National Laboratory Animal Center (Taipei, Taiwan). Rats were housed under a 12-h light–dark cycle at 22-24°C with a relative humidity of 65-70%. After one-week adaptation, rats were randomly divided into four groups (*n* =10 per group): control, CCl_4_, CCl_4_ + ginseng extract (GE) and CCl_4_ + ginsenoside Rb1 (Rb1) groups. The normal diet based on Laboratory Rodent Diet 5001 powder was purchased from PMI Nutrition International Inc. (Brentwood, MO). Ginseng extract (Ashland Inc., Covington, KY, USA) containing 800 g ginsenosides/kg extract (80%) (ginsenosides in the extract include Rb1, Rc, Rd, Rg1, Rg2, Rg3, Rh1 and Rh2) and ginsenoside Rb1 (China Chemical & Pharmaceutical Co., Ltd., Taipei, Taiwan) with 98% purity were blended with the normal diet at a dose of 0.5 g/kg and 0.05 g/kg, respectively. Ginsenoside Rb1 content was equal in the GE and Rb1 groups. Rats were fed ginseng extract or ginsenoside Rb1 two weeks before (week 0, W0) the induction of liver injury by intraperitoneal injection of 400 ml/l CCl_4_ in olive oil at a dose of 0.75 ml/kg body weight weekly for 7 weeks. The control group was injected with an equal volume of olive oil without CCl_4_. Food intake, water intake and body weight were recorded throughout 9-week experimental period. This study was approved by the Institutional Animal Care and Use Committee of Taipei Medical University.

### Histopathological examination

After 9 weeks, rats were euthanized with ether and liver samples from left lateral lobe, median lobe and right lateral lobe were collected for histopathological and biochemical analyses. Excised liver specimens from different lobes (1 cm × 1 cm) were fixed in 10% paraformaldehyde, embedded in paraffin, sectioned and stained with hematoxylin and eosin (H&E), Masson’s trichrome or silver. The specimens were coded with a single-blind method and graded from 0 (no lesion), 1 (trace lesion), 2 (weak lesion), 3 (moderate lesion) to 4 (severe lesion) for fat changes, and from 0 (no lesion), 1 (lesion in the central vein area), 2 (lesion in the central vein area and expansion to the surrounding area) to 3 (lesion in the central and portal vein areas or cirrhosis) for necrosis, inflammation, and fibrosis under a light microscope by a pathologist.

### Plasma alanine aminotransferase (ALT) and aspartate aminotransferase (AST) activities

Blood samples from rat tails were collected into heparin-containing tubes at weeks 0, 2 (CCl_4_ injection) and 9. Blood was centrifuged at 3000 *g* for 15 min at 4°C. Plasma ALT and AST activities were measured spectrophotometrically at 570 nm using a commercial kit (RM 163-K, Iatron Laboratories Inc., Tokyo, Japan).

### Plasma and hepatic lipid concentrations

Blood samples from rat tails were collected at weeks 0, 2 and 9, and centrifuged at 3000 *g* for 15 min at 4°C. Liver samples from left lateral lobe, median lobe and right lateral lobe were homogenized in chloroform/methanol (2:1) solution and extracted by chloroform/methanol/water (3:48:47) solution. Triglycerides and total cholesterol concentrations in plasma and liver were determined spectrophotometrically at 500 nm using commercial enzymatic kits (Randox® TR213 for triglycerides, Randox® CH201 for total cholesterol, Randox Laboratories Ltd., London, UK).

### Hepatic inflammatory markers

Liver slices (0.5 g) were homogenized in 1.5 mL of buffer solution (50 mmol/l Tris, 150 mmol/l NaCl, and 10 ml/l Triton X-100, pH 7.2) [20] and mixed with 100 μl of proteinase inhibitor cocktail solution (P8340, Sigma-Aldrich, Inc., Saint Louis, USA). Liver homogenate was centrifuged at 3000 *g* for 15 min at 4°C for TNF-α, IL-1β and IL-10 analysis. For PGE_2_ and soluble ICAM-1 (sICAM-1) analysis, liver slices (0.5 g) were mixed with 1.0 ml of homogenized buffer (0.25 mol/l sucrose, 50 mmol/l Tris–HCl, and 5 mmol/l EDTA, pH 7.5). Liver homogenate was centrifuged at 8000 *g* for 15 min at 4°C.

Hepatic TNF-α, IL-1β, IL-10, PGE_2_ and sICAM-1 levels were measured spectrophotometrically using enzyme-linked immunosorbent assay (ELISA) kits (Quantikine® RTA00 for TNF-α, Quantikine® RLB00 for IL-1β, DuoSet® DY522 for IL-10, PGE_2_, Quantikine® KGE004 for PGE_2,_ Quantikine® RIC100 for sICAM-1, R&D Systems, Inc., Minneapolis, USA). Hepatic supernatant was separately incubated with rat anti-TNF-α, anti-IL-1β, anti-IL-10, anti-PGE_2_ or anti-sICAM-1, then washed with wash buffer (0.05% Tween® in phosphate buffer solution, PBS) followed by incubation with polyclonal antibody against TNF-α, IL-1β, PGE_2_ or sICAM-1 conjugated to horseradish peroxidase or biotinylated anti-IL-10 secondary antibody with streptavidin conjugated to horseradish peroxidase, respectively. After washed with wash buffer several times, the substrate solution (hydrogen peroxide and chromogen tetramethylbenzidine) was added and the reaction was terminated by adding diluted hydrochloric acid. The absorbance was determined at 450 nm. Protein concentration was measured by the method of Lowry *et al*. [21].

### Hepatic hydroxyproline, MMP-2 and TIMP-1 levels

Hepatic hydroxyproline level was measured by colorimetric assay. Freeze-dried liver specimen (0.25 g) was homogenized with 2 ml of distilled water. Liver homogenate was hydrolyzed in alkaline solution (2 mol/L NaOH), oxidized with chloramines T reagent, and incubated with Ehrlich’s reagent at 65°C. The chromogenic product was determined spectrophotometrically at 550 nm.

The levels of MMP-2 and TIMP-1 in the liver were determined by commercial kits (Quantikine® DMP200 for MMP-2, Quantikine® RTM100 TIMP-1, R&D Systems, Inc.) using ELISA. Liver slices were homogenized with PBS and proteinase inhibitor cocktail solution and centrifuged at 12000 *g* for 10 min at 4°C. The supernatant was centrifuged again and collected for further analysis. Hepatic supernatant was separately incubated with rat anti-MMP-2 or anti- TIMP-1, washed with wash buffer, and incubated with polyclonal antibody against MMP-2 or TIMP-1 conjugated to horseradish peroxidase followed by several washes with wash buffer. The substrate solution (hydrogen peroxide and chromogen tetramethylbenzidine) was added for the reaction and stop solution (diluted hydrochloric acid) was then added to stop the reaction. The absorbance was measured at 450 nm.

### Statistical analysis

All data were expressed as mean ± SD. The data were analyzed by one-way analysis of variance (ANOVA) using Statistical Analysis System (SAS version 9.1, SAS Institute Inc., Cary, NC, USA). The difference between any two groups was analyzed by Fisher’s least significant difference test. A value *p* <0.05 was considered significant.

## Results

### Body weight, liver weight and food intake

The results of body weight, liver weight and food intake were shown in Table 1 to monitor the effects of the treatments on gross growth and liver weight. Final body weight and weight gain were significantly higher in the control group than those in the CCl_4_ (*p* <0.01), GE (*p* <0.05), and Rb1 (*p* <0.05) groups (Table 1), but not significantly different among the three CCl_4_ treated groups. Daily intake of ginseng extract and ginsenoside Rb1 was 12.6 ± 0.6 mg (33.8 ± 1.5 mg/kg body weight) and 1.3 ± 0.6 mg (3.3 ± 0.1 mg/kg body weight) in the GE and Rb1 groups, respectively. The relative liver weight was significantly higher in the CCl_4_ and GE groups than that in the control group (*p* <0.01). The Rb1 group significantly reduced the relative liver weight compared with the CCl_4_ group (*p* <0.05). However, total liver weight and daily food intake did not differ significantly among the four groups.

**Table 1:** **Body weight, liver weight and food intake in rats treated with ginsenosides against CCl**_**4**_ **-induced liver damage**

	Control	CCl _4_	GE	Rb1
Initial body weight (g)	240 ± 8^a^	233 ± 12^a^	236 ± 14^a^	239 ± 15^a^
Final body weight (g)	478 ± 23^b^	427 ± 25^a^	433 ± 38^a^	448 ± 37^a^
Weight gain (g)	238 ± 22^b^	194 ± 23^a^	208 ± 34^a^	209 ± 30^a^
Total liver weight (g)	15.9 ± 2.5^a^	17.5 ± 1.5^a^	17.3 ± 3.8^a^	16.4 ± 1.8^a^
Relative liver weight (g/kg)	33.2 ± 4.8^a^	41.1 ± 3.4^c^	39.7 ± 5.8^bc^	36.7 ± 3.4^ab^
Food intake (g/d)	26.5 ± 1.6^a^	25.3 ± 1.0^a^	25.2 ± 1.2^a^	25.4 ± 1.2^a^

Data are presented as mean ± SD (n =10). Values not sharing the same superscript differ significantly (*p* <0.05) within the same row.

### Histopathological examination

**Figure 1:**
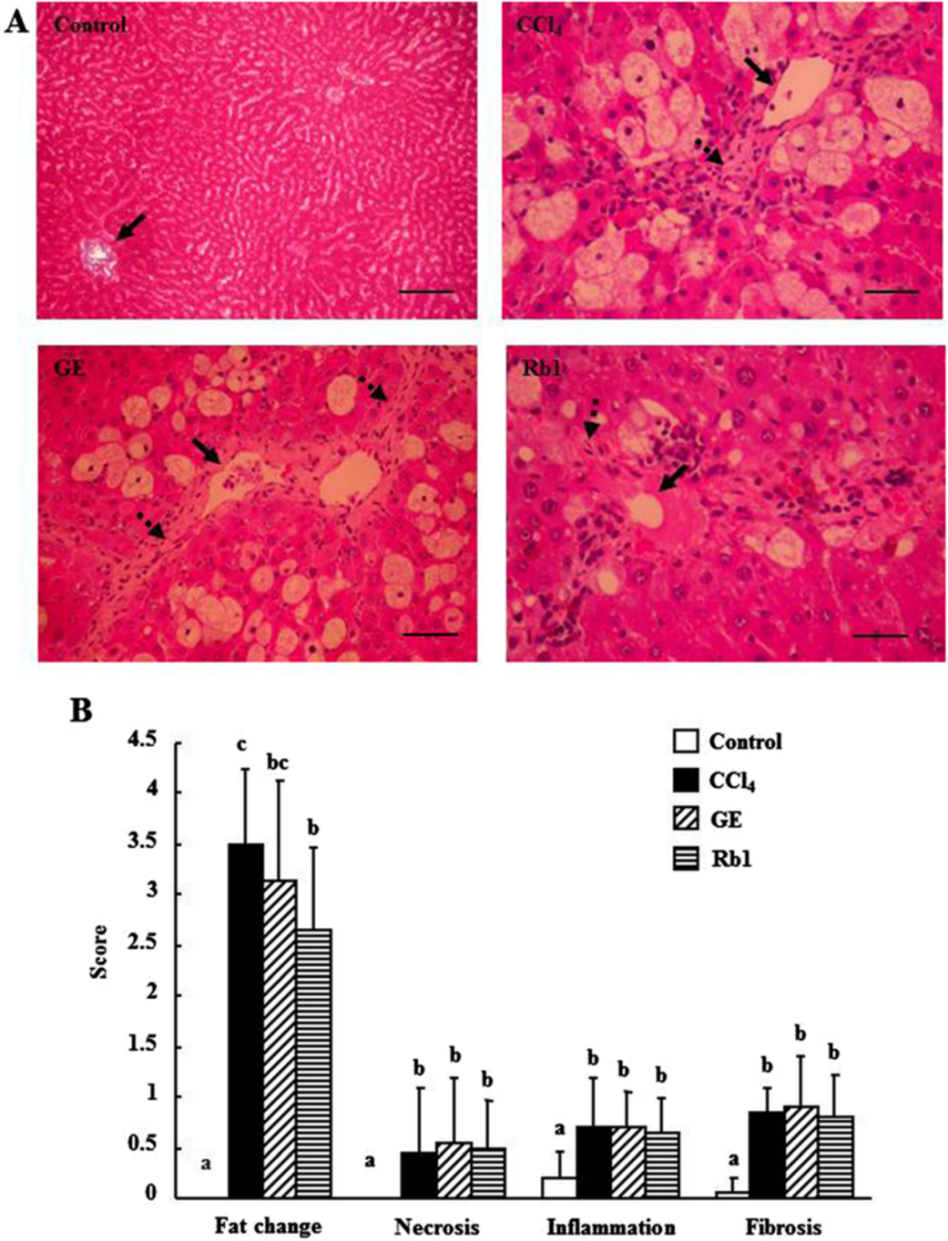
**The representative histological sections of rat liver specimens. A**: hematoxylin and eosin stain at 20 × 10 magnification, **B**: semi-quantitative scores graded from 0 (no lesion), 1 (trace lesion), 2 (weak lesion), 3 (moderate lesion) to 4 (severe lesion) for fat changes, and from 0 (no lesion), 1 (lesion in the central vein area), 2 (lesion in the central vein area and expansion to the surrounding area) to 3 (lesion in the central and portal vein areas or cirrhosis) for necrosis, inflammation and fibrosis in control, CCl_4_, GE and Rb1 groups. Solid and dashed arrows represent the central vein and collagen fibers. Data are presented as mean ± SD (n =10). Values not sharing the same letter differ significantly (*p* <0.05). Scale bar =50 μm.

**Figure 2:**
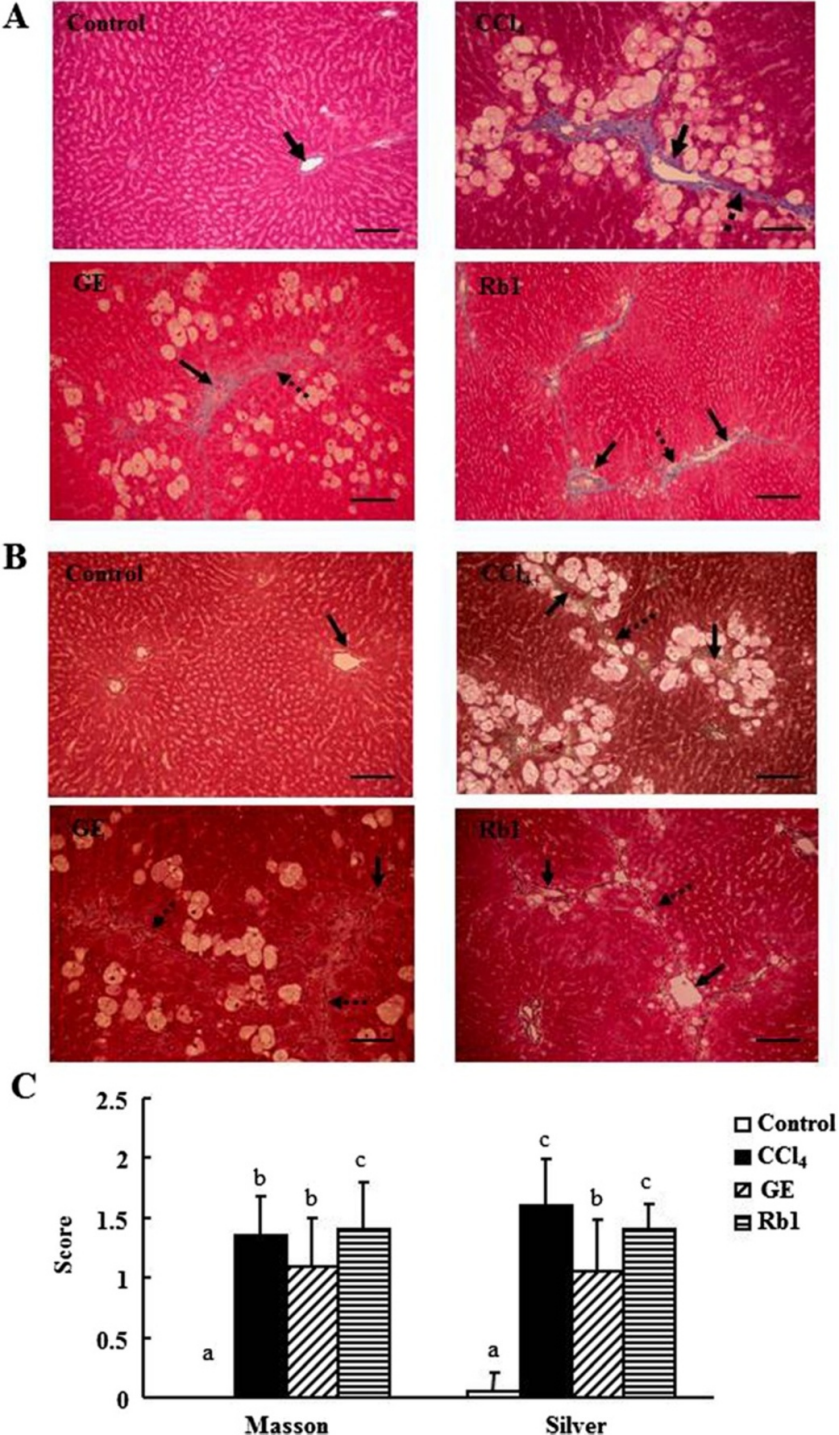
**The representative histological sections of rat liver specimens. A**: Masson’s trichrome stain at 15 × 10 magnification, **B**: silver stain at 15 × 10 magnification; **C**: semi-quantitative scores graded from 0 (no collagen formation), 1 (collagen formation in the central vein area), 2 (collagen and fibrous bridge formation in different central vein areas) to 3 (cirrhosis) for fibrosis in the control, CCl_4_, GE and Rb1 groups. Collagen fibers stained with Masson’s trichrome appear blue. Reticular fibers stained with silver appear brown. Solid and dashed arrows represent the central vein and fiber bridging. Data are presented as mean ± SD (n =10). Values not sharing the same letter differ significantly (*p* <0.05). Scale bar =50 μm.

The results of the histopathological examination by different stains were demonstrated in Figures 1 and 2 to determine the effects of the treatments on histopathological changes in the liver, especially on liver fibrosis. The bright red color of H&E staining shown in Figure 1A could be resulted from strong eosin staining, a fluorescent red dye. The pathological sections stained by H&E showed that no fat was accumulated in the liver of the control group, whereas large fat vacuoles were observed in the liver of the CCl_4_ group (Figure 1A). However, the Rb1 group had significantly decreased fat vacuoles compared with the CCl_4_ group (2.65 ± 0.82 vs. 3.50 ± 0.75, *p* <0.05) (Figure 1B). The pathological scores for fat change were not significantly different between the GE and Rb1 groups. The CCl_4_, GE, and Rb1 groups had significantly elevated cell necrosis (*p* <0.05), inflammatory cells (*p* <0.01), and fibrosis (*p* <0.01) in the central veins compared with the control group. However, the pathological scores for necrosis, inflammation, and fibrosis in the liver did not significantly differ among the three CCl_4_ treated groups.

The pathological assessment of liver fibrosis as observed by Masson’s trichrome stain demonstrated that the formation of collagen fibers appeared blue was elevated by the exposure to CCl_4_ (Figure 2A). The fibrosis scores determined by Masson’s trichrome stain in the CCl_4_ (1.35 ± 0.34), GE (1.10 ± 0.39) and Rb1 (1.40 ± 0.39) groups were significantly higher compared with the control group (*p* <0.01) (Figure 2C), but the GE group had a lower liver fibrosis score compared with the Rb1 group (*p* <0.05). The accumulation of liver reticular fibers stained by silver and appeared brown was increased by the exposure to CCl_4_ (*p* <0.01) (Figure 2B). The GE (1.05 ± 0.44) group had significantly reduced accumulation of reticular fibers than the CCl_4_ (1.60 ± 0.39, *p* <0.01) and Rb1 (1.40 ± 0.21, *p* <0.05) groups (Figure 2C).

### Plasma ALT and AST activities

Plasma ALT and AST activities were measured to assess the effects of the treatments on liver functions. Plasma ALT and AST activities were significantly elevated in the CCl_4_ group than those in the control, GE and Rb1 groups (*p* <0.01) after the induction of liver injury (W2) (Table 2). The CCl_4_ group still had increased plasma ALT (*p* <0.05) and AST (*p* <0.01) activities compared with the control group, whereas plasma ALT and AST activities did not differ among the control, GE and Rb1 groups at week 9. The GE group significantly decreased plasma AST activity compared with the CCl_4_ group at week 9 (*p* <0.05).

**Table 2:** **Plasma ALT and AST activities in rats treated with ginsenosides**

	Control	CCl _4_	GE	Rb1
ALT activity (IU/l)				
W0	29.6 ± 2.9^a^	27.6 ± 1.7^a^	31.2 ± 3.8^a^	29.2 ± 32.8^a^
W2	33.2 ± 3.6^a^	4012.4 ± 2212.9^b^	469.0 ± 435.2^a^	1072.6 ± 618.6^a^
W9	33.6 ± 4.9^a^	334.9 ± 379.1^b^	136.4 ± 167.4^ab^	177.5 ± 333.0^ab^
AST activity (IU/l)				
W0	75.9 ± 6.3^a^	78.3 ± 4.4^a^	70.8 ± 23.5^a^	77.6 ± 5.3^a^
W2	62.1 ± 20.2^a^	8370.4 ± 5360.8^b^	2155.0 ± 1973.8^a^	1233.6 ± 616.7^a^
W9	63.3 ± 6.0^a^	288.4 ± 181.3^b^	134.9 ± 114.6^a^	171.3 ± 227.9^a^

Data are presented as mean ± SD (n =10). Values not sharing the same superscript differ significantly (*p* <0.05) within the same row.

### Plasma and hepatic triglyceride and total cholesterol concentrations

Plasma and hepatic lipid concentrations were determined to evaluate the effects of the treatments on lipid profiles. After the induction of liver injury (W2), plasma triglycerides were significantly increased and still maintained higher level at week 9 in the CCl_4_ group compared with those in the control group (*p* <0.01) (Table 3). Treatment with GE (*p* <0.01) and Rb1 (*p* <0.05) significantly reduced plasma triglycerides compared with the CCl_4_ group to the similar level of the control group at weeks 2 and 9.

**Table 3:** **Plasma triglycerides and total cholesterol concentrations in rats treated with ginsenosides**

	Control	CCl _4_	GE	Rb1
Triglycerides (mmol/l)				
W0	0.16 ± 0.04^a^	0.16 ± 0.04^a^	0.14 ± 0.03^a^	0.15 ± 0.02^a^
W2	0.24 ± 0.04^a^	0.39 ± 0.19^b^	0.25 ± 0.04^a^	0.28 ± 0.10^a^
W9	0.54 ± 0.10^c^	0.35 ± 0.06^b^	0.27 ± 0.05^a^	0.29 ± 0.07^a^
Total cholesterol (mmol/l)				
W0	0.90 ± 0.11^b^	0.71 ± 0.12^a^	0.79 ± 0.16^ab^	0.75 ± 0.10^a^
W2	1.01 ± 0.14^a^	1.20 ± 0.18^b^	1.05 ± 0.21^ab^	0.95 ± 0.27^a^
W9	0.79 ± 0.11^a^	0.70 ± 0.17^a^	0.67 ± 0.10^a^	0.66 ± 0.20^a^

Data are presented as mean ± SD (n =10). Values not sharing the same superscript differ significantly (*p* <0.05) within the same row.

At the baseline, plasma total cholesterol level was significantly lower in the CCl_4_ and Rb1 groups than that in the control group (*p* <0.01). After the induction of liver injury, total cholesterol level was significantly elevated in the CCl_4_ group than that in the control and Rb1 groups (*p* <0.05) (Table 3). Plasma total cholesterol level did not differ significantly among the four groups at week 9.

**Figure 3:**
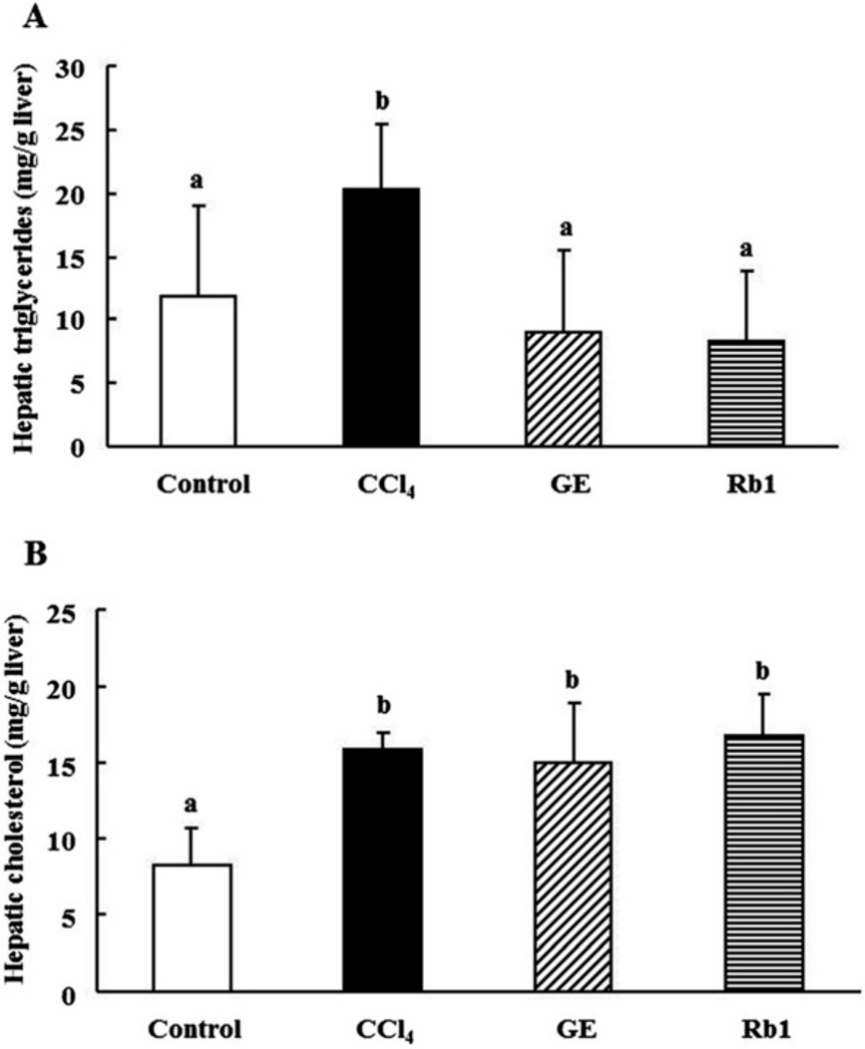
**Effects of ginsenosides extract and ginsenoside Rb1 on hepatic lipids in rats. A**: hepatic triglycerides, **B**: total cholesterol concentration. Data are presented as mean ± SD (n =10). Values not sharing the same letter differ significantly (*p* <0.05).

Hepatic triglyceride concentrations were significantly increased by 73% in the CCl_4_ group than those in the control group (*p* <0.01), and decreased by 56% and 60% in the GE and Rb1 groups, respectively, compared with the CCl_4_ group (*p* <0.01) (Figure 3A). Hepatic total cholesterol level was significantly greater in the CCl_4_, GE and Rb1 groups than that in the control group (*p* <0.01), but not significantly different among CCl_4_ treated groups (Figure 3B).

### Hepatic TNF-α, IL-1β, IL-10, PGE_2_, and sICAM-1 levels

The results of hepatic cytokine levels were found in Table 4 to determine the effects of the treatments on hepatic mediators released in the inflammatory condition. Hepatic IL-1β (*p* <0.01), PGE_2_ (*p* <0.05) and sICAM-1 (*p* <0.05) levels were significantly elevated, whereas hepatic IL-10 level was significantly decreased in the CCl_4_ group compared with those in the control group (Table 4). Hepatic TNF-α, IL-1β and PGE_2_ levels were significantly reduced in the GE group compared with the CCl_4_ group (*p* <0.05). The Rb1 group had higher hepatic TNF-α level than the GE group, but lower hepatic PGE_2_ level than the CCl_4_ group (*p* <0.05).

**Table 4:** **Hepatic TNF-α, IL-1β, IL-10, PGE**_**2**_ **, and sICAM-1 levels in rats treated with ginsenosides**

	Control	CCl _4_	GE	Rb1
TNF-α (pg/mg protein)	15.8 ± 7.6^ab^	17.2 ± 5.1^b^	10.4 ± 3.3^a^	16.7 ± 6.9^b^
IL-1β (pg/mg protein)	132.5 ± 39.6^a^	333.0 ± 135.8^c^	212.0 ± 115.2^ab^	292.9 ± 182.9^bc^
IL-10 (pg/mg protein)	4461 ± 958^b^	3443 ± 508^a^	3513 ± 677^a^	3514 ± 600^a^
PGE_2_ (pg/mg protein)	6196 ± 1599^b^	9822 ± 2610^c^	4636 ± 1928^ab^	4128 ± 1480^a^
sICAM-1 (pg/mg protein)	2782 ± 771^a^	3991 ± 867^b^	3288 ± 567^ab^	3427 ± 963^ab^

Data are presented as mean ± SD (n =10). Values not sharing the same superscript differ significantly (*p* <0.05) within the same row.

### Hepatic hydroxyproline, MMP-2 and TIMP-1 levels

**Figure 4:**
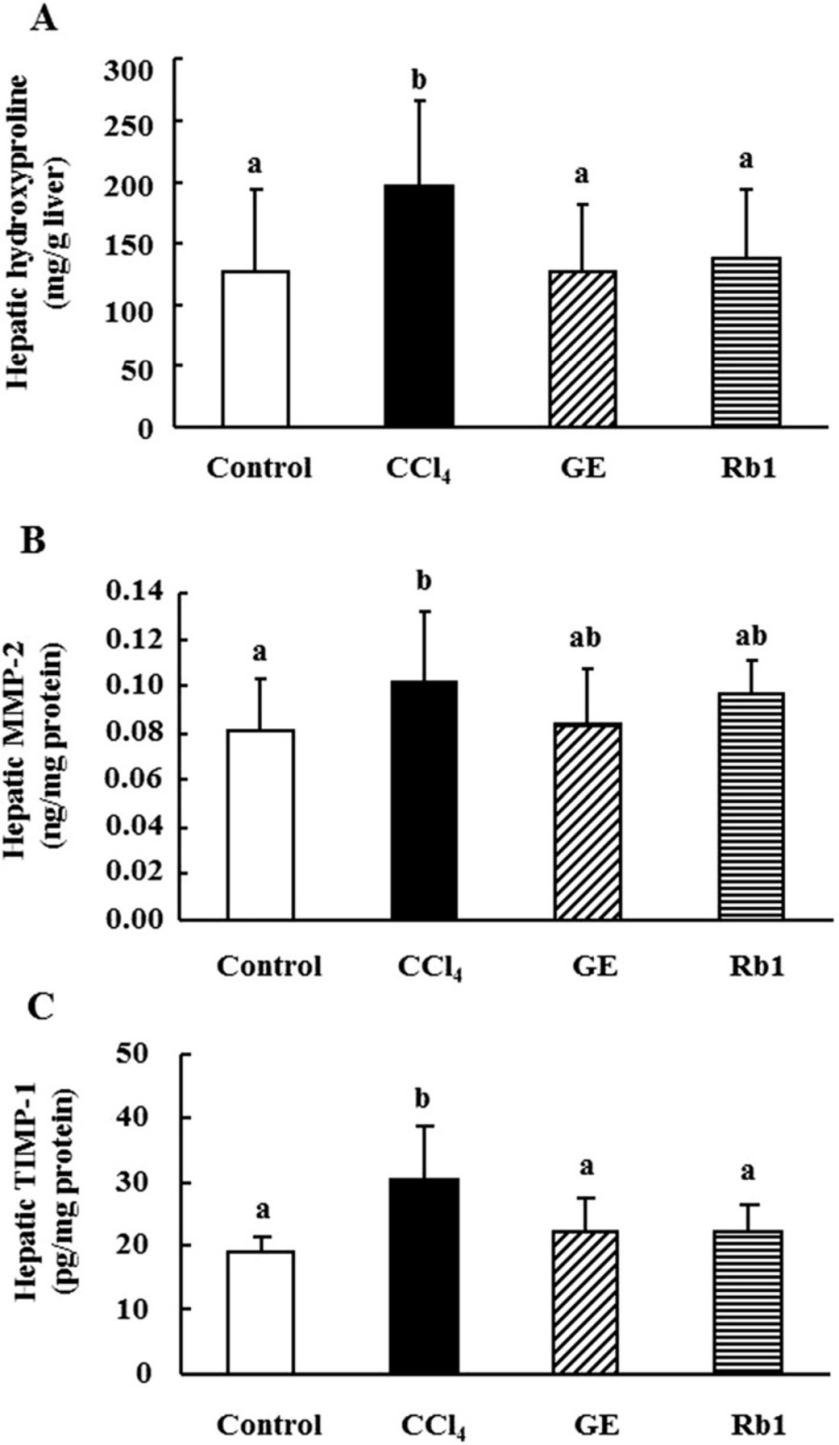
**Effects of ginsenosides extract and ginsenoside Rb1 on liver fibrosis markers in rats. A**: hepatic hydroxyproline level, **B**: MMP-2 level; **C**: TIMP-1 level. Data are presented as mean ± SD (n =10). Values not sharing the same letter differ significantly (*p* <0.05).

The results of hepatic hydroxyproline, MMP-2 and TIMP-1 levels were shown in Figure 4 to investigate the effects of the treatments on liver fibrogenesis and fibrolysis. Hepatic hydroxyproline (*p* <0.05), MMP-2 (*p* <0.05) and TIMP-1 (*p* <0.01) levels were elevated by 55%, 28% and 61%, respectively, in the CCl_4_ group compared with the control group (Figure 4). Ginseng extract and ginsenoside Rb1 treatments significantly reduced hepatic hydroxyproline level by 36% and 30% (Figure 4A) and TIMP-1 level by 27% and 27% (Figure 4C), respectively, compared with the CCl_4_ group (*p* <0.05). However, hepatic MMP-2 level was not different in the three CCl_4_ treated groups (Figure 4B).

## Discussion

Similar to the previous study [22], plasma ALT and AST activities were increased by CCl_4_-induced liver injury. Ginseng extract and ginsenoside Rb1 significantly decreased plasma ALT and AST activities elevated by the exposure to CCl_4_. The previous studies demonstrated that ginseng extract or heated ginseng exhibited antioxidant activity and acted as a free radical scavenger to inhibit lipid peroxidation *in vitro* and increase catalase and superoxide dismutase activities in V79-4 lung fibroblast cells [14, 23]. Moreover, ginsenoside Rb1, Rg1 or derived metabolite-compound K decreased hepatic malondialdehyde level and increased serum ALT and AST activities [16, 18]. Therefore, ginseng extract and ginsenoside Rb1, as a free radical scavenger, may eliminate free radical damage to the hepatocytes.

Exposure to CCl_4_ led to significant increases in the accumulation of fat vacuoles and the levels of triglycerides and total cholesterol in the liver. The abnormal fat accumulation in the liver caused by CCl_4_ could be attributed to: (1) the imbalance between lipogenesis and lipolysis by increasing lipid synthesis and the rate of lipid esterification [24] as well as by decreasing cAMP production via the stimulation of hormone-sensitive lipase [25, 26], and (2) impaired synthesis and secretion of very low density lipoprotein through the interference of glycosylation and maturation of lipoglycoprotein by free radicals which are produced by CCl_4_ metabolism [24, 27], or through the inactivation of Ca^+2^-ATPase pump in the mitochondria and endoplasmic reticulum [6, 28].

Liver damage and elevated hepatic triglycerides induced by CCl_4_ were improved by the treatment of GE and Rb1. Red ginseng saponin, containing ginsenosides Rb1, Rb2, Rc, Rd, Re and Rg1, played a crucial role in hepatoprotection by suppressing oxidative stress and lipid peroxides via inhibiting the expression and activity of cytochrome P450 in the liver [29]. Consistent with our findings, ginsenoside Rb1 injected intraperitoneally at a dose of 10 mg/kg body weight for 3 d significantly decreased hepatic lipids by increasing hepatic cAMP production [30]. Additionally, Rb1 injected intraperitoneally at a dose of 10 mg/kg body weight also showed to reduce hepatic triglyceride accumulation in high fat diet-induced obese rats by increasing hepatic carnitine palmitoyltransferase 1 activity and cellular AMP/ATP ratio to stimulate fatty acid oxidation and suppress lipogenesis, respectively [31]. Compound K, a major intestinal metabolite of ginsenosides, has been demonstrated to elevate gene expression of peroxisome proliferator-activated receptor-α and decrease gene expression of fatty acid synthase and stearoyl-CoA desaturase 1 through activating AMP-activated protein kinase in HepG2 human hepatoma cells [32]. The previous study revealed that ginseng extract rich in ginsenosides suppressed hepatic cholesterol synthesis via inhibiting hepatic β-hydroxy-β-methylglutaryl-CoA reductase and cholesterol 7α-hydroxylase activities [33]. These results suggest that ginseng extract, ginsenoside Rb1 and their metabolite may accelerate lipid utilization and suppress lipid biosynthesis in the liver to further decrease elevated hepatic triglycerides induced by CCl_4_ exposure.

Kupffer cells activated by oxidative stress secrete cytokines, such as TNF-α and IL-1β, to stimulate the expression of sICAM-1 which induces the activation of neutrophils [7]. The results of H&E staining showed accumulation of inflammatory cells in the CCl_4_ group. Furthermore, fibrotic bridges were observed in the histopathological sections stained by silver in the CCl_4_ group as cells became necrotic and reticular fibers resembled after frameworks collapsed. Ginseng extract reduced accumulation of reticular fibers, ameliorated cell necrosis and inhibited production of TNF-α and IL-1β. In agreement with our present study, *in vitro* studies demonstrated that ginseng and ginsenoside Rb1 suppressed TNF-α production and IL-1β mRNA expression in murine RAW264.7 macrophages [34, 35]. Ginsenoside Rg1, one of the important components in *P. ginseng*, intravenously injected at 20 mg/kg body weight significantly attenuated serum TNF-α and IL-6 release in septic mice [36].

The expression of PGE_2_ and cycloogenase-2 (COX-2) are induced by inflammatory response, and the expression of COX-2 was stimulated by proinflammatory cytokines, such as TNF-α and IL-1β [37]. Our present study found that ginseng extract and ginsenoside Rb1 significantly decreased hepatic PGE_2_ level induced by CCl_4_. It is presumed that ginseng extract and ginsenoside Rb suppressed PGE_2_ production through reducing proinflammatory cytokines and suppressing COX-2 expression. Furthermore, activation of NF-κB modulates expression and secretion of proinflammatory cytokines, chemokines, adhesion molecules, COX-2 and inducible nitric oxide synthase (iNOS) [38]. Ginsenoside Rb1, Rg1, Rg3, Rh1 and their derived metabolite compound K down-regulated activation of NF-κB and simultaneously suppressed PGE_2_, ICAM-1, COX-2 and iNOS expression *in vitro* [17, 19, 39–42]. It is presumed that ginseng extract and ginsenoside Rb1 attenuated production of proinflammatory factors possibly via inhibiting NF-κB activation.

The accumulation of hydroxyproline and collagen fibers was found in the CCl_4_ group, whereas ginseng extract and ginsenoside Rb1 decreased hepatic hydroxyproline and TIMP-1 levels to inhibit liver fibrosis. Oxidative stress induced by CCl_4_ metabolism could further stimulate proliferation and invasiveness of hepatic stellate cells (HSCs) [43]. Proliferated HSCs resulted in increases in TGF-β1 secretion, which activates HSCs and induces gene expression of type I collagen, and induction of collagen accumulation. Activated HSCs express MMPs and their tissue inhibitors (TIMPs). Oxidative stress stimulated MMP-2 production by HSCs via extracellular signal-regulated kinase1/2 and phosphatidylinositol 3 kinase pathways [43], and MMPs induce HSCs proliferation and migration [44]. *In vitro* studies found that ginsenoside Rb1 inhibited HSCs activation and mRNA expression of type I and III collagen, TGF-β1 and TIMP-1 [45], and its metabolite induced apoptosis in HSCs via caspase-3 activation pathway [46]. Compound K was found to inhibit MMP-2 expression and NF-κB activation in the *in vitro* model [47]. Ginsenoside Rg1 subcutaneously injected at 50 and 100 mg/kg body weight attenuated serum levels of hyaluronic acid and type III procollagen, and hepatic hydroxyproline level in rats with thioacetamide-induced liver fibrosis [48]. The decrease in activated HSCs could lead to inhibition of fibrogenesis and TIMP-1 expression by reducing TNF-α and TGF-β1 [49]. Our finding demonstrated that ginseng extract and ginsenoside Rb1 diminished hepatic TIMP-1 level accompanied with decreased TNF-α level. Therefore, ginsen*g* extract and ginsenoside Rb1 could suppress activation and proliferation of HSCs and further inhibit liver fibrosis.

Ginsenoside Rb1 content was equivalent in the GE and Rb1 groups in the present study. Ginseng extract with additional seven ginsenosides except for ginsenoside Rb1 more effectively diminished collagen accumulation and inhibited TNF-α production compared with ginsenoside Rb1. Therefore, the hepatoprotective and anti-inflammatory actions of ginseng extract on CCl_4_-induced liver damage could be attributed to the synergistic action of overall ingredients including the remaining 20% constituents and their metabolites.

## Conclusions

In conclusion, *Panax ginseng* extract (0.5 g/kg) and ginsenoside Rb1 (0.05 g/kg) decrease plasma ALT and AST activities elevated by CCl_4_-induced liver damage and inhibit the accumulation of triglycerides in the liver. The levels of TNF-α, PGE_2_, hydroxyproline and TIMP-1 in the liver are diminished by ginseng extract and ginsenoside Rb1. Therefore, ginseng extract and ginsenoside Rb1 attenuate CCl_4_-induced liver injury through anti-inflammatory and antifibrotic effects.

## Abbreviations

ALT: Alanine aminotransferase
AST: Aspartate aminotransferase
CCl_4_: Carbon tetrachloride
COX-2: Cycloogenase-2
GE: Ginseng extract
HSCs: Hepatic stellate cells
IL: Interleukin
iNOS: Inducible nitric oxide synthase
MMP: Matrix metalloproteinase
NF-κB: Nuclear factor-κB
NO: Nitric oxide
PGE_2_: Prostaglandin E_2_
sICAM-1: Soluble intercellular adhesion molecule-1
TGF-β: Transforming growth factor-β
TIMPs: Tissue inhibitor of metalloproteinases
TNF-α: Tumor necrosis factor-α.
